# Cell Engineering and Cultivation of Chinese Hamster Ovary Cells for the Development of Orthogonal Eukaryotic Cell-free Translation Systems

**DOI:** 10.3389/fmolb.2022.832379

**Published:** 2022-04-14

**Authors:** Jeffrey L. Schloßhauer, Niño Cavak, Anne Zemella, Lena Thoring, Stefan Kubick

**Affiliations:** ^1^ Branch Bioanalytics and Bioprocesses (IZI-BB), Fraunhofer Institute for Cell Therapy and Immunology (IZI), Potsdam, Germany; ^2^ Institute of Chemistry and Biochemistry, Freie Universität Berlin, Berlin, Germany; ^3^ Faculty of Health Sciences, Joint Faculty of the Brandenburg University of Technology Cottbus –Senftenberg, The Brandenburg Medical School Theodor Fontane and the University of Potsdam, Potsdam, Germany

**Keywords:** orthogonal translation, cell-free protein synthesis, CRISPR, amber suppression, *E. coli* tyrosyl-tRNA synthetase, *M. mazei* pyrrolysyl-tRNA synthetase, membrane protein, C12orf35

## Abstract

The investigation of protein structures, functions and interactions often requires modifications to adapt protein properties to the specific application. Among many possible methods to equip proteins with new chemical groups, the utilization of orthogonal aminoacyl-tRNA synthetase/tRNA pairs enables the site-specific incorporation of non-canonical amino acids at defined positions in the protein. The open nature of cell-free protein synthesis reactions provides an optimal environment, as the orthogonal components do not need to be transported across the cell membrane and the impact on cell viability is negligible. In the present work, it was shown that the expression of orthogonal aminoacyl-tRNA synthetases in CHO cells prior to cell disruption enhanced the modification of the pharmaceutically relevant adenosine A2a receptor. For this purpose, in complement to transient transfection of CHO cells, an approach based on CRISPR/Cas9 technology was selected to generate a translationally active cell lysate harboring endogenous orthogonal aminoacyl-tRNA synthetase.

## Introduction

The production of proteins represents an important building block to elucidate the fundamental biochemical signatures of cells and forms the basis for diverse industrial applications. Therefore, further development of appropriate production systems is of particular interest. Cell-based systems are commonly exploited to address questions in basic research and to meet the demand for biotechnologically relevant proteins such as drug targets. Proteins can be equipped with special properties or new reactive groups for broader applications. Although conjugation of proteins *via* cysteine residues with sulfhydryl chemical groups or coupling of lysine residues with N-hydroxysuccinimide esters is widely used, the ligation of the reacting amino acids is not homogeneously distributed and resulted in a heterogeneous protein mixture (Shadish and DeForest, 2020). In addition, there are reports of intein-based protein ligation and conjugation of proteins by cysteine transpeptidases (Debelouchina and Muir, 2017; Vogl et al., 2021). However, protein sequence motifs located at the N- or C- terminus must be incorporated into the protein of interest to ensure appropriate protein modification. In contrast, genetic code expansion strategies have the advantage that amino acid positions in proteins can be modified site-specifically (Chung et al., 2020). This requires an orthogonal aminoacyl-tRNA synthetase (aaRS)-tRNA pair, which does not cross-react with endogenous aaRS, tRNAs and amino acids, while maintaining high specificity towards the non-canonical amino acids (ncaa). Usually, a desired sense codon in the protein will be changed to an amber stop codon, recognized by the aminoacylated orthogonal tRNA or the release factor, resulting in either amber suppressed or truncated proteins (Beyer et al., 2020). In this process, ncaa with various chemical groups such as azides, alkynes and strained alkenes can be incorporated into the protein of interest, enabling specific reactions based on azide-alkyne cycloadditions, Staudinger ligation as well as tetrazine ligation (Kenry and Liu, 2019; Mushtaq et al., 2019). Moreover, various ncaa could be incorporated into proteins, such as photocrosslinking amino acids, NMR sensitive probes and amino acids, which were equipped with post-translational modifications like phosphorylation (Chin et al., 2002; Jones et al., 2010; Rogerson et al., 2015). Albeit orthogonal systems are utilized for protein modifications *in vivo*, ncaa must be supplemented to the culture medium at high concentrations as uptake into cells is limited, thus imposing a burden on the cells (Takimoto et al., 2010; Lin et al., 2017). Furthermore, overexpression of pharmaceutical proteins including membrane proteins can lead to decreased cell viability and growth, thereby limiting these systems for further applications (Gubellini et al., 2011). On the one hand, an alternative production system based on translationally active cell lysates, offers the possibility of producing proteins such as toxins and membrane proteins that can negatively affect cell growth and viability (Khambhati et al., 2019). Due to their open character, cell-free protein synthesis (CFPS) systems in general can be supplemented by various additives, making it feasible to realize protein modifications based on orthogonal translation systems (Lu, 2017). Previous work on *Sf*21 cell-free protein synthesis systems showed that the modified EGF receptor, which is misregulated in various tumors, was translocated into microsomal structures, originating from the endoplasmic reticulum (Quast et al., 2016; Thoring et al., 2017). In the process described by Quast et al., the ncaa p-azido-L-phenylalanine (AzF) was integrated at different amino acid positions based on the orthogonal mutant *E. coli* tyrosyl-tRNA synthetase (eAzFRS)-tRNAtyr pair. Kapoor et al. demonstrated conjugation of cell-free synthesized malaria surface antigen Pfs25 to a GPI linker derivate and following immunization of mice, leading to higher anti-Pfs25 antibody titers with Pfs25-GPI compared to Pfs25 (Kapoor et al., 2018).

The cell-free system can be further improved by directly overexpressing desired proteins in host cell-lines used for the generation of cell lysate. It has been shown that expression of T7 RNA polymerase in *E. coli* eliminates the need for supplementation of the subsequent cell-free reaction (Des Soye et al., 2019). In a previous study, orthogonal pyrrolysyl-tRNA synthetase (PylRS) was expressed prior to cell disruption of *E. coli* and followed by the incorporation of two unnatural lysine derivatives into EGFP in an orthogonal CFPS reaction (Chemla et al., 2015).

In contrast to frequently used *E. coli* systems, protein expression in eukaryotic systems is often more complex but necessary to provide proteins with post-translational modifications such as glycosylation, disulfide bond formation and lipidation (Gillette et al., 2019; Tripathi and Shrivastava, 2019). Approximately 70% of clinically approved proteins are produced in Chinese hamster ovary (CHO) cells due to their robustness, growth to high cell densities, high protein yields and the ability to synthesize complex mammalian proteins (Jayapal et al., 2007). Nowadays, protein expression is often based on stable transfected cells using sequence-specific recombinases and transposon-based modifications as well as RNA-guided nuclease based integration techniques, rather than random integration methods (Matasci et al., 2011; Zhang et al., 2015; Grav et al., 2017). Of particular importance is the clustered regularly interspaced short palindromic repeats (CRISPR)/Cas9 technology for genome editing in mammalian cells, which now makes it possible, to specifically knock out genes and insert expression cassettes at well-defined positions into the genome with high efficiency (Jinek et al., 2012; Ran et al., 2013). For protein overexpression, transcriptionally active loci in the genome that are less affected by gene silencing are preferentially targeted. To date, various loci including *HPRT*, *H11*, *COSMC*, *Rosa26* and *C12orf35* were addressed to insert desired transgenes into the CHO genome (Lee et al., 2015; Gaidukov et al., 2018; Kawabe et al., 2018; Zhao et al., 2018; Chi et al., 2019).

**FIGURE 1:**
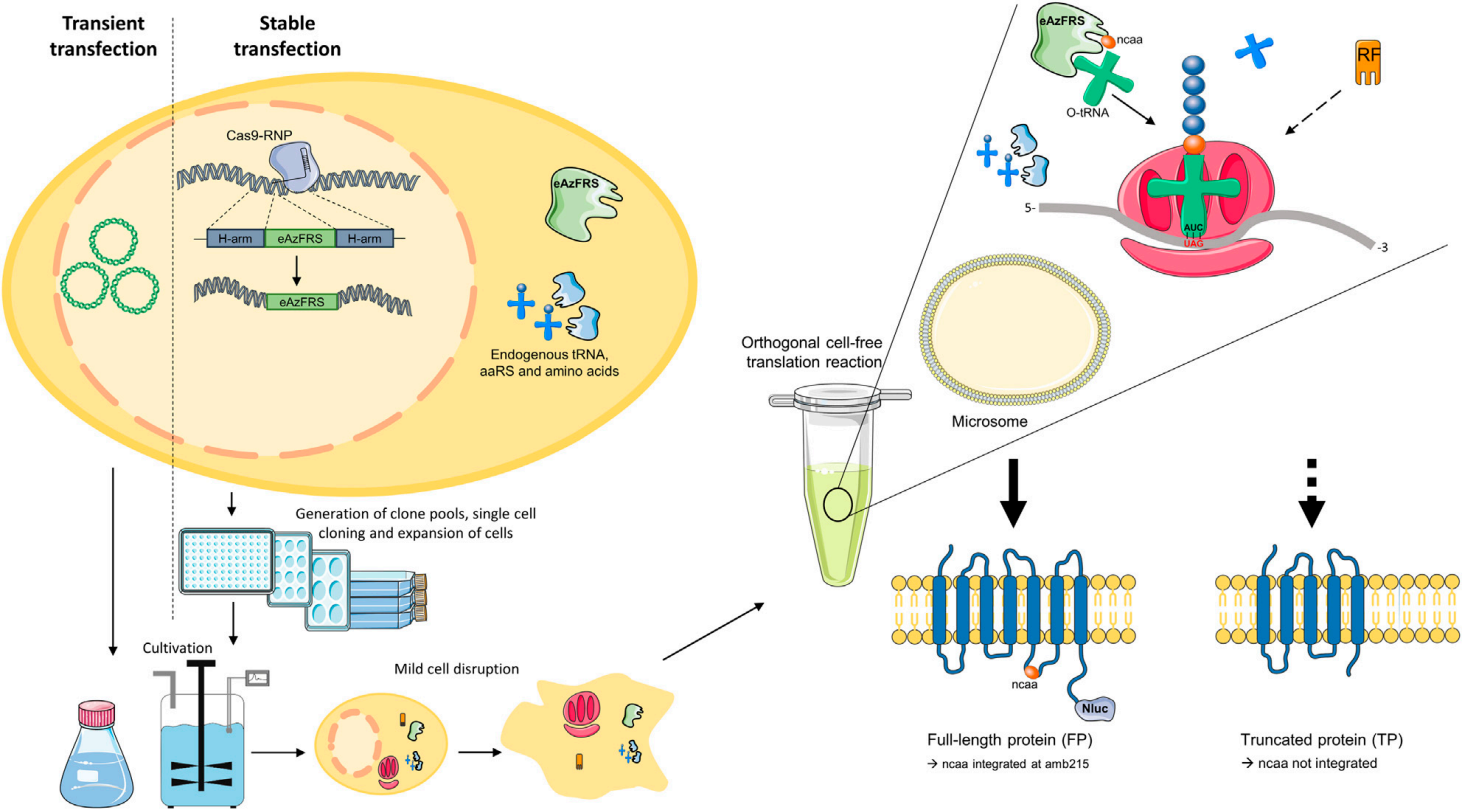
General workflow. CHO cells were either transiently or stably transfected. While transiently transfected CHO cells were cultured in shake flasks and bioreactors for two days, stable transfected cells were subjected to selection pressure to obtain clone pools and clonal cell lines. The adenosine A2a receptor, which is C-terminally coupled to a Nanoluciferase (Nluc), was used to evaluate the developed orthogonal modification system. In the presence of orthogonal eAzFRS and orthogonal tRNA (o-tRNA), as well as the non-canonical amino acid (ncaa) p-azido-L-phenylalanine, the amber stop codon UAG at amino acid position 215 can be addressed to produce amber suppressed full-length protein (FP). Upon binding of the eukaryotic release factor (RF) termination product (TP) will be produced. Arrows indicate the direction of the workflow. The dashed arrow indicates translation termination. Parts of the figure were created by using pictures from Servier Medical Art (http://smart.servier.com/). Servier Medical Art by Servier is licensed under a Creative Commons Attribution 3.0 Unported License.

The objective of this study was to determine whether orthogonal aaRS can be expressed in CHO cells without diminishing the translational activity of the resulting cell lysate for CFPS. To this end, the orthogonal eAzFRS was transiently transfected into CHO cells and convenient cultivation formats were examined to attain a cell lysate optimal for modifying the G-protein-coupled adenosine A2a receptor in cell-free reactions. We also attempted to develop an orthogonal CFPS system based on stable transfection of eAzFRS into the CHO genome by targeting the *HPRT1* and *C12orf35* loci through CRISPR technology. A general workflow of the presented study is shown in Figure 1.

## Materials and Methods

### Plasmids

The plasmids A2aRamb and A2aR for cell-free synthesis of adenosine A2a receptor (with and without an amber stop codon at amino acid position 215) were utilized as previously described (Zemella et al., 2019). A Nanoluciferase sequence was linked to the C-terminus of the receptor. The plasmid for cell-free synthesis of the modified *E.coli* tyrosyl-tRNA-synthetase (eAzFRS) in an *E.coli* based cell-free reaction was previously described (Zemella et al., 2019). The utilized PylRS-AF coding sequence contained the mutations Y306A and Y384F as previously reported (Yanagisawa et al., 2008). Transient transfection of CHO cells was performed with pcDNA3.1(+)-eAzFRS and pcDNA3.1(+)-PylRS-AF expression vectors. A Strep-Tag was fused to the C-terminus of eAzFRS, while a 6xHis-Tag was fused to the N-terminus of the PylRS-AF. For stable transfection of CHO cells the plasmid pSpCas9(BB)-2A-GFP (Plasmid #48138 from Addgene) was used to express Cas9 enzyme linked to GFP by a 2A self-cleavage peptide. The guide RNA sequences gRNA-HPRT1-T1: 5′-GTA​GAA​TGA​TCA​GTC​AAC​AG-3′ and gRNA-C12orf35-T1: 5′-GCC​CCT​TAC​AGC​TGT​AGA​TA-3′ targeting the *C12orf35* and *HPRT1* locus in the CHO genome were extracted from the literature (Zhao et al., 2018). Novel gRNA sequences gRNA-HPRT1-T2: 5′-GGG​GTT​GTA​CCG​CTT​GAC​CA-3′ and gRNA-C12orf35-T2: 5′-GCC​GGG​ACT​TAA​CCA​CTC​GA-3′ were designed with the CRISPOR gRNA design tool. Gibson assembly was used to clone the gRNA sequences into the gRNA_Cloning Vector (Plasmid #41824 from Addgene) according to the protocol previously described (Mali et al., 2013). The donor vector sequence for stable transfection was designed similar to a previously described study on CHO cells (Zhao et al., 2018). Therefore, a human cytomegalovirus promoter was used to drive gene expression of the GFP or eAzFRS gene and a bovine growth hormone (BGH)-polyA sequence for termination of transcription was used. The expression cassette for the resistance gene puromycin was under the control of the phosphoglycerate kinase-1 promoter and BGH-polyA utilized to terminate transcription. The expression cassette was flanked by locus (*HPRT1* or *C12orf35*)-specific homology arms. Additionally, gRNA sequences were located at the ends of the homology arms to create a linear donor in presence of Cas9 enzyme (Supplementary Figure S1).

### Cell Culture

CHO-K1 cells were provided from the Leibniz Institute DSMZ-German Collection of Microorganisms and Cell Cultures GmbH (DSMZ no: ACC110) and adapted to grow in suspension. CHO-K1 cells were cultured in serum-free ProCHO5 medium (Lonza) at 37°C, 5% CO_2_ and 100 rpm. Cells were seeded at densities of 1 × 10^6^ cells/ml and grown up to 5 × 10^6^ cells/ml in shake flasks, unless otherwise noted. Cell growth was monitored by counting cells using trypan blue staining and a Luna-FL cell counter (Logos Biosystems, Gyeonggi-do, Korea).

### Transient Transfection

CHO cells were transiently transfected with eAzFRS and PylRS-AF plasmids by polyethylenimine (PEI) reagent. Therefore, cells were grown to 2 × 10^6^ cells/ml and plasmid was added in a ratio of 1.5 µg/10^6^ cells, followed by 2 µg PEI reagent/10^6^ cells. Afterwards cells were incubated for four hours rotating at 37°C and 5% CO_2_ and diluted to obtain a cell density of 1 × 10^6^ cells/ml in 1,000 ml final volume in either 2 L shake flasks or 1 L bioreactor vessels (Biostat B-DCUII, Sartorius Stedim Biotech GmbH). CHO cells were cultivated for two days and harvested for cell lysate preparation as described previously (Thoring and Kubick, 2018).

### Stable Transfection and Single-Cell Cloning

Plasmid DNA was mixed in a ratio of 2:2:1 (eAzFRS donor vector: gRNA vector: Cas9 vector) to obtain 500 ng plasmid DNA in 5 µL PBS. The DNA was further diluted by addition of 100 µL Opti-MEM serum-free medium (Gibco). Lipid based transfection was conducted by combine the diluted plamsid DNA with 2.5 µL Lipofectamine LTX (Thermo Fisher Scientific) and 0.5 µL Plus reagent. After incubation for 30 min at room temperature the transfection mixture was added to 1 × 10^6^ cells/ml in 500 µL serum-free ProCHO5 medium (Lonza) in a 24-well plate. Cells were mixed gently and centrifuged for 15 min at room temperature and 400xg to increase transfection efficiency similar to a previous report (Barbu and Welsh, 2007). Following 48 h at 37°C, 5% CO_2_ without shaking, cells were selected by puromycin resistance. Therefore, a concentration of 10 µg/ml puromycin in culture medium was achieved for two weeks. Positively transfected cells were pre-selected by array dilution, while maintaining the puromycin selection pressure. Clone pools were isolated and analyzed by qPCR and submitted to single-cell cloning by limiting dilution.

### Fluorescent Microscopy

The GFP fluorescence of stable transfected CHO cells was visualized by a Nikon Eclipse TS2 inverted microscope combined with the NIS-Elements imaging software. A combination of a fluorescent LED and a GFP-B filter cube was used, whereby the filter cube is composed of a 470/40 nm excitation filter, a 500 nm dichroic filter and a 535 nm barrier filter. Images were recorded with a Nikon DS-Fi3 camera system.

### Genotyping PCR

Genomic DNA was extracted from 10^6^ CHO cells by the Quick-DNA Miniprep Plus Kit (Zymo Research) according to the manufacturer’s instruction. Following genotyping primer sequences flanking the integration side of the expression cassette in the *C12orf35* locus were designed: GT-C12-T2-FW 5′-TGC​ATG​CAC​CAC​AGA​GTC​AT-3′ and GT-C12-T2-RV 5′-ACA​GGG​CGC​TTT​GAT​GGT​AA-3´. Genotyping PCR was performed by combining 0.5 µM of each primer, 5 ng/µL genomic DNA, 1x HotStar HiFidelity PCR Buffer (Qiagen), 0.05 U/µl HotStar HiFidelity DNA Polymerase (Qiagen) and distilled water in a 20 µL reaction. The following temperature profile was used: 95°C for 5 min; 40 cycles of 94°C for 15 s, 62°C for 60 s, 68°C for 5 min; 72°C for 10 min. PCR-products were run on a 1% agarose gel and product size was analyzed by comparing them to the Quick-Load 2-Log DNA Ladder (0.1–10.0 kbp, New England Biolabs).

### Quantitative PCR

Quantitative real-time PCR (qPCR) was used to determine the eAzFRS mRNA levels. Therefore, mRNA was extracted with the High Pure RNA Isolation Kit (Roche) according to the manufacturer’s instruction. Afterwards cDNA was prepared by using the Transcriptor First Strand cDNA Synthesis Kit (Roche). qPCR-primer sequences qRT-Gnb1-FW 5′-CCA​TAT​GTT​TCT​TTC​CCA​ATG​GC-3′ and qRT-Gnb1-RV 5′-AAGTCGTCGTACCCAGCAAG-3′of the housekeeping gene G protein subunit beta 1 (Gnb1) were extracted from the literature (Brown et al., 2018). The primer sequences qRT-AzFRS-FW 5′-GGA​TAA​GAA​CAG​CGG​CAA​GG-3′ and qRT-AzFRS-RV 5′-TCC​ATC​TCC​ACC​ATA​GGC​AC-3′ were used for eAzFRS amplification. The qPCR reaction mix consisted of 0.5 µM qPCR primers, 2x FastStart Essential DNA Green master-mix (Roche), 10 ng cDNA and water in a 20 µL reaction. qPCR reactions were analyzed by the LightCycler 96 System (Roche). The temperature profile of the qPCR was 95°C for 5 min, followed by 38 cycles at 95°C for 20 s, 63°C for 20 s and 72°C for 20 s. A melt curve analysis was performed to confirm the specificity of amplification. Therefore, samples were heated for 5 min at 95°C and cooled down to 60°C. Afterwards the samples were heated continuously at 0.1°C/s. Relative quantification was calculated using Gnb1 as reference gene and the sample with the lowest gene expression as a control according to the Pfaffl method (Pfaffl, 2001). Measurements were conducted in triplicate and data were expressed as the mean with standard deviation.

### Bioreactor Based Cultivation

Initial cell densities of 0.7 × 10^6^ cells/ml, unless otherwise noted, were achieved in a 1 L bioreactor vessel connected to the control unit (Biostat B-DCUII, Sartorius Stedim Biotech GmbH). The cells were cultured while pH and oxygen supply were kept constant until a cell density of approx. 3–7 × 10^6^ cells/ml was reached. During fermentation the parameters stirrer speed, pH, pO_2_ and cultivation temperature were tracked over time and cell counts were measured regularly. The batch process was repeated up to three cycles, while maintaining a defined initial cell density in each cycle.

### Cell-free Protein Synthesis

CHO cell lysate for CFPS was prepared to contain endogenous microsomes, taking advantage of the endoplasmic reticulum, as previously described in detail (Brödel et al., 2014; Thoring and Kubick, 2018). Eukaryotic cell-free reactions were composed of 40% CHO cell lysate, 10 µM PolyG, 30 mM HEPES-KOH (pH 7.5, Carl Roth GmbH), 100 mM sodium acetate (Merck), 3.9 mM magnesium acetate (Merck), 150 mM potassium acetate (Merck), 100 µM amino acids (Merck), 250 µM spermidin (Roche), 2.5 mM Dithiothreitol (Life technologies GmbH), 100 µg/ml creatine phosphokinase (Roche), 20 mM creatine phosphate (Roche), 1.75 mM ATP (Roche) and 0.3 mM GTP (Roche). To enable the qualitative analysis of radio-labeled proteins by electrophoresis 30 µM radioactive ^14^C-leucine (specific radioactivity 46.15 dpm/pmol, Perkin Elmer) was added to the reaction. Furthermore, 1 U/μl T7 RNA polymerase (Agilent), 0.3 mM of UTP (Roche), 0.3 mM CTP (Roche) and 0.1 mM of the cap analogue m7G(ppp)G (Prof. Edward Darzynkiewicz, Warsaw University, Poland) were added to the reaction to implement transcription and translation reactions simultaneously. Orthogonal cell-free reactions based on eAzFRS were further supplemented with 4 µM orthogonal tRNA_Tyr_ and 2 mM p-Azido-L-phenylalanine (AzF), while PylRS-AF based reactions were supplemented with 4 µM orthogonal tRNA_Pyl_ and 2 mM strained cyclooctyne (SCO). Reactions containing purified eAzFRS were supplemented with 4 µM eAzFRS unless otherwise noted. Reactions were incubated for three hours at 30°C and 600 rpm. The eAzFRS and the *in vitro* transcribed tRNAtyr were prepared as previously described in detail (Zemella et al., 2019). Briefly, the eAzFRS was synthesized in a cell-free reaction based on *E. coli* lysate using the RTS 500 E.coli HY Kit (biotechrabbit). Purification of eAzFRS was performed using Strep-Tactin Superflow high capacity columns (IBA Life Sciences) due to the presence of a C-terminal Strep tag II within the coding sequence of eAzFRS.

### Autoradiography of Radiolabeled Proteins

The crude reaction mixture was precipitated after CFPS by acetone. Therefore, 3 µL of reaction mix was diluted with 47 µL distilled water followed by three times the sample volume of cold acetone. After incubation on ice for 15 min samples were centrifuged 10 min at 16,000 × g. Supernatants were discarded while precipitated proteins were dried for 30 min at 45°C to get rid of remaining acetone. Afterwards, dried protein pellets were resuspended in LDS-sample buffer (Invitrogen) containing 50 mM DTT. Samples were mixed at 1,400 rpm at room temperature. Denaturing polyacrylamide gel electrophoresis (PAGE) was carried out with NuPAGE 10% Bis-Tris Gels. Gels were stained and dried by a vacuum chamber (Unigeldryer 3545D, Uniequip), deposited on phosphor screens (GE Healthcare) for three days and screens were scanned by an imaging system (Typhoon TRIO + Imager, GE Healthcare). The presence of ^14^C leucine in CFPS allowed the visualization of synthesized proteins based on autoradiography.

### Luciferase Assay

The analysis of Nanoluciferase (Promega) activity was performed by utilizing the Nano-Glo Luciferase Assay System (Promega). Therefore, 50 µL of the assay reagent was mixed with 3 µL crude mix of a cell-free reaction and luminescence was detected by the Multimode Microplate Reader Mithras^2^ LB 943 (Berthold Technologies GmbH) by using an OD2 filter. Measurements were conducted in triplicate and data were expressed as the mean with standard deviation.

### Semi-Quantitative Western Blot

5 × 10^5^ CHO cells were lysed for 30 min on ice using RIPA lysis buffer (10 mM Tris–HCl pH 8, 140 mM NaCl, 1% Triton X-100, 1 mM EDTA, 0.5 mM EGTA, 0.1% SDS, 0.1% sodium deoxycholate), supplemented with cOmplete-ULTRA protease inhibitor cocktail (Roche). The supernatant was isolated after 10 min centrifugation (16,000 × g) at 4°C. The protein concentration was determined by the Pierce BCA Protein Assay Kit (Thermo Fisher Scientific) and 2.5 µg proteins were heated at 70°C for 10 min in LDS sample buffer (Invitrogen). Samples were separated by denaturing polyacrylamide gel electrophoresis with NuPAGE 10% Bis-Tris Gels. Protein transfer to a PVDF membrane was carried out by the iBlot Dry Blotting System (Invitrogen). The membrane was blocked with 5% bovine serum albumin (BSA). The C-terminally located Strep-tag II of the eAzFRS coding sequence allowed western blot analysis by using anti-Strep II (ab76949, Abcam) as primary antibody. Furthermore, anti-Actin (I-19) (sc-1616, Santa Cruz Biotechnology) was utilized as primary antibody since β-Actin served as the internal control. Primary antibodies were diluted 1:1,000 in TBS with 0.1% Tween 20 and 5% BSA. Detection of signals was enabled by using the anti-rabbit IgG, HRP-linked antibody (#7074, Cell Signaling Technology) as secondary antibody (1:3,000 in TBS with 0.1% Tween 20 and 5% BSA). After detection of eAzFRS, removal of antibodies from the blot membrane was achieved by incubating the membrane at 50°C for 30 min in stripping buffer (0.5 M Tris HCl pH 6.8, 10% SDS, 0.8% 2-mercaptoethanol). Following detection of β-Actin was performed on the stripped PVDF membrane. Band intensities of eAzFRS signals were normalized to β-Actin signals.

### Data and Statistical Analysis

The amber suppression efficiency was calculated by dividing the relative light unit (RLU) value based on cell-free reactions containing the A2aRamb construct by the RLU value based on cell-free reactions containing the A2aR construct without an amber stop codon. After multiplying the ratio by 100 the amber suppression efficiency was expressed as a percentage. The experimental data were expressed as mean values ± standard error, with “n” representing the number of biological replicates if not otherwise specified. Differences between three independent experiments were analyzed using a Welch’s *t*-test. The maximum growth rate of analyzed cell lines was calculated from the slope of log-transformed data. Therefore, non-linear fitting of cell growth data was performed by using the SRichards1 function of Origin (Pro) software, Version Number 2019 (OriginLab Corporation, Northampton, MA, United States).

## Results

### Expression of eAzFRS in CHO Cells Prior to Cell Lysate Preparation Enables Orthogonal Cell-Free Reactions

**FIGURE 2:**
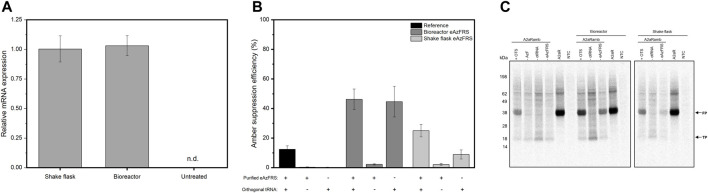
CFPS based on transiently transfected CHO cells expressing eAzFRS. **(A)** Relative eAzFRS mRNA expression levels two days after transfection. Experiments were performed in technical triplicates and bars represent the standard deviation of the mean. **(B)** Amber suppression efficiency based on cell-free synthesized A2aR and A2aRamb constructs using reference cell lysates and cell lysates from transiently transfected CHO cells. Samples with a plus indicate the addition of either 4 µM purified eAzFRS and orthogonal tRNA, while a minus indicates the absence of the particular orthogonal component. Luminescence signals of cell-free reactions without a template (NTC) were subtracted from sample signals prior to calculation of suppression efficiency. Data are shown as mean ± SEM (*n* = 3). **(C)** Autoradiography of electrophoretically separated proteins based on a cell-free reaction with A2aR, A2aRamb constructs and a no-template control (NTC). n.d.: not detected, FP: Full-length protein, TP: Truncated protein, otRNA: Orthogonal tRNA, AzF: p-Azido-L-phenylalanine, OTS: Orthogonal translation system, TrCHO: Transiently transfected CHO cells.

A previous study on orthogonal translation based on *E.coli* cell-free systems showed that transformation of *E.coli* with the *Methanosarcina mazei* orthogonal pyrrolysyl-tRNA synthetase prior to cell disruption circumvent time-intensive purification steps, leading to an optimized orthogonal cell-free protein synthesis system (Chemla et al., 2015). Based on this, we aimed to transiently transfect CHO cells with eAzFRS to create an eukaryotic cell-free system with the ability to site-specifically modify difficult-to-express proteins, such as the membrane protein adenosine receptor A2a (A2aR). Additionally, the goal was to identify optimal cultivation conditions, while keeping transfection conditions constant, for producing a highly active CHO cell lysate enabling site-specific labeling of proteins in CFPS. Transiently transfected cells were either cultured in a shake flask or in a bioreactor to evaluate the influence of the culture format on cell lysate activity. Transcriptional analysis by quantitative PCR (qPCR) revealed similar eAzFRS mRNA levels for both cultivation formats (Figure 2A). As expected, mRNA expression could not be detected in non-treated cells due to the absence of endogenous eAzFRS. CHO cells were harvested two days post-transfection to ensure sufficient protein expression prior to cell lysate preparation. Characterization of orthogonal aaRS activity of the resulting processed cell extracts was achieved by evaluating cell-free synthesis of a reporter gene construct composed of the GPCR coding sequence containing an amber stop codon at position 215 and a C-terminal Nanoluciferase (Nluc) and is referred to as A2aRamb (Zemella et al., 2019). Once, the amber stop codon is suppressed by orthogonal tRNA-ncaa, the C-terminal Nluc activity can be correlated to the aminoacylation activity based on the orthogonal aaRS, since the luminescent output was shown to be linear (Supplementary Figure S2). A2aR construct without an amber stop codon was utilized to assess the efficiency of ncaa incorporation into the desired protein, referred to as amber suppression efficiency. A concentration of 4 µM purified eAzFRS was determined to be optimal in cell-free reactions based on CHO lysate (Supplementary Figure S3).

To exclude the possible negative effect of PEI reagent on cell lysate and thus on CFPS, a reaction set-up was included that was based on cell lysate derived from CHO cells transfected only with PEI reagent without any DNA template (Supplementary Figure S4). Unexpectedly, cell lysate based on CHO cells treated only with PEI resulted in higher protein expression compared to non-treated CHO cells, which may be caused by batch-to-batch variations affecting cell lysate efficiency. Considering the comparable resulting translationally active cell lysate of PEI treated CHO cells, cell lysates processed from transfected cells in a 1 L bioreactor and 2 L shake flask were analyzed for orthogonal translation in CFPS. A reference lysate based on non-treated CHO cells was utilized as a control. CFPS by using the reference lysate shows no translation after reaching the stop codon if omitting either orthogonal tRNA (otRNA) or purified eAzFRS, while combining orthogonal components led to a suppression efficiency of 12% (Figure 2B). In contrast, without addition of purified eAzFRS to cell-free reactions based on novel lysates, a higher amber suppression efficiency of 45% for bioreactor and 9% for shake flask based cultivations was detected. When supplementing cell-free reactions with purified eAzFRS, we came across further increase of suppression efficiencies up to 46% (bioreactor) and 25% (shake flask). Qualitative electrophoretic analysis of proteins from cell-free reactions with A2aRamb and A2aR supplemented with ^14^C-leucine showed expression of full-length proteins (FP) and termination products (TP) as expected (Figure 2C). Overall, these results demonstrate the functionality of the orthogonal system by expressing the eAzFRS in CHO cells prior to cell lysate preparation.

### Integration of eAzFRS Into the CHO Genome Creates a Robust System for Orthogonal Cell-Free Reactions

One should acknowledge the heterogeneous expression and the fact that eAzFRS expression only persists for a limited time if cells are transiently transfected. Here we conduct an alternative approach to generate an *in vitro* orthogonal translation system by utilizing CHO cells stably transfected with eAzFRS. Several studies have demonstrated the use of CRISPR/Cas technology to overexpress a diverse repertoire of proteins from defined genomic loci in CHO cells (Lee et al., 2015; Eisenhut et al., 2018; Iwao et al., 2021). The genomic loci *HPRT1* and *C12orf35* were investigated for the generation of stable CHO cell lines using CRISPR/Cas, as reported in a recent study (Zhao et al., 2018). According to this, we have aimed to target respective loci to integrate an eAzFRS expression cassette into the CHO genome. Therefore, we analyzed four different gRNAs to modify the *HPRT1* and *C12orf35* locus of the CHO genome (Table 1). Either locus was targeted with one gRNA (referred to as gHPRT1-T1 and gC12-T1), which was previously identified (Zhao et al., 2018). Additionally, two gRNAs were designed that were predicted to have a higher specificity score value and less off-target effects and were referred to as gHPRT1-T2 and gC12-T2.

**TABLE 1:** Utilized guide RNA sequences

gRNA	Locus	Origin
gHPRT1-T1	*HPRT1*	gRNA from Zhao et al. (2018)
gHPRT1-T2	*HPRT1*	Novel gRNA
gC12-T1	*C12orf35*	gRNA from Zhao et al. (2018)
gC12-T2	*C12orf35*	Novel gRNA

**FIGURE 3:**
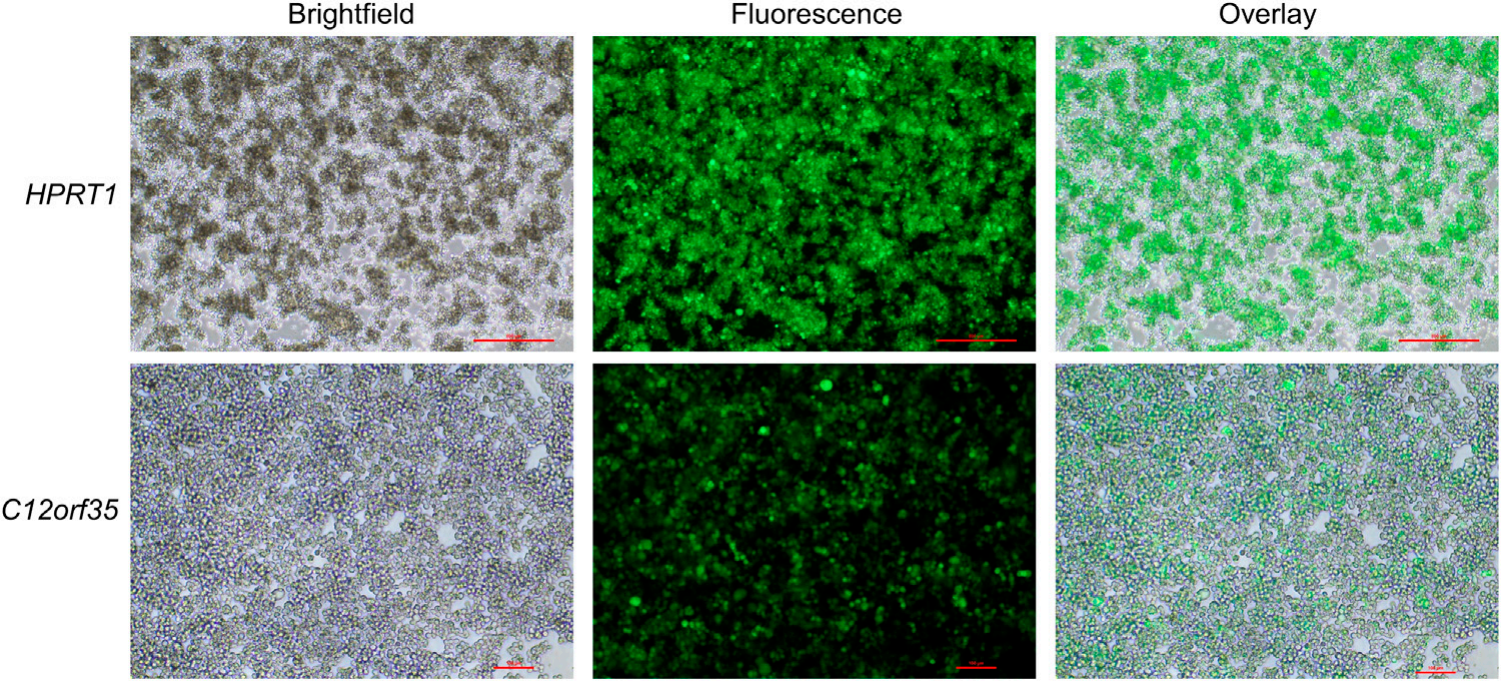
CHO cells stable transfected with GFP. CHO cells were stable transfected by CRISPR/Cas9 to integrate GFP into the *HPRT1* or *C12orf35* locus. Cells were subjected to puromycin selection pressure to enrich positively transfected cells. Microscopic images of GFP clone pools are shown. Scale bars for *HPRT1* measure 500 μm, while scale bars for *C12orf35* measure 100 μm.

**FIGURE 4:**
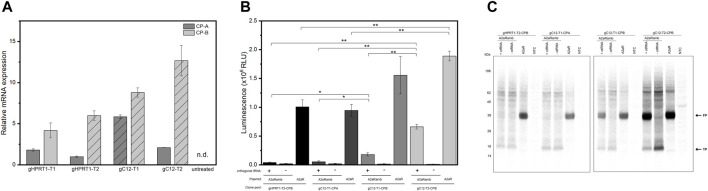
CHO clone pools stable transfected with eAzFRS. **(A)** Relative eAzFRS mRNA expression levels of CHO clone pools. The line pattern indicates selected clone pools for cell-free synthesis. Experiments were performed in technical triplicates and bars represent the standard deviation. **(B)** Luciferase assay based on cell-free synthesized A2aR and A2aRamb constructs using cell lysates based on clone pools gHPRT1-T2-CPB, gC12-T1-CPA, gC12-T1-CPB and gC12-T2-CPB. Samples with a plus or minus indicate the addition or absence of orthogonal tRNA, respectively. Luminescence signals of cell-free reactions without a template (NTC) were subtracted from sample signals. Data are shown as mean ± SEM (*n* = 3). A Welch’s *t*-test was performed to determine the statistically significant differences between samples (*p-value < 0.05; **p-value < 0.01). **(C)** Exemplary autoradiography of electrophoretically separated proteins of a CFPS based on the different clone pools. CP: Clone pool, n.d: not detected, otRNA: Orthogonal tRNA, FP: Full-length protein, TP: Truncated protein, NTC: No-template control.

First, stable transfection of CHO cells was conducted by using a GFP expression cassette targeting either *HPRT1* or *C12orf35* to examine whether the locations were suitable for expression. Afterwards selection pressure (puromycin) was applied. Qualitative microscopic analysis demonstrated GFP expression of genetically modified cells (Figure 3). Both target sites showed a high proportion of enriched fluorescent cells, leading us to integrate an eAzFRS expression cassette into both loci. Selection of stable transfected cells was conducted by supplementation of puromycin. Two promising CHO cell clone pools for each gRNA target were evaluated by qPCR. The relative mRNA expression of eAzFRS is shown in Figure 4A. The change in gene expression is relative to the sample with the lowest gene expression. A 8.8-fold and 12.7-fold higher mRNA expression could be observed for the clone pools (CP) B by exploiting gC12-T1 and gC12-T2, respectively, compared to the lowest expression by using gHPRT1-T2 (CP-A). Further investigation on clone pools (marked in Figure 4A) by producing cell lysates highlighted their performance in orthogonal cell-free reactions. Therefore, three different cell lysates of gHPRT1-T2-CPB, gC12-T1-CPA, gC12-T1-CPB and gC12-T2-CP-B were generated and the eAzFRS activity was evaluated by the C-terminal Nluc activity. Indeed, significant higher luminescence could be observed for clone pool gC12-T2-CP-B compared to the other examined clone pools in cell-free reactions with the A2aRamb construct (Figure 4B). Furthermore, significant higher signals were achieved for clone pool gC12-T2-CP-B compared to gHPRT1-T1-CPB and gC12-T1-CPA using the construct A2aR without an amber position, indicating a generally higher protein production. Thus clone pool gC12-T2-CP-B was identified as the most promising candidate for subsequent isolation of CHO clones. Electrophoretic analysis supported the observed data of the luciferase assay (Figure 4C).

### Single Clone Selection Circumvents Clonal Variations and Ensures Reproducibility

**FIGURE 5:**
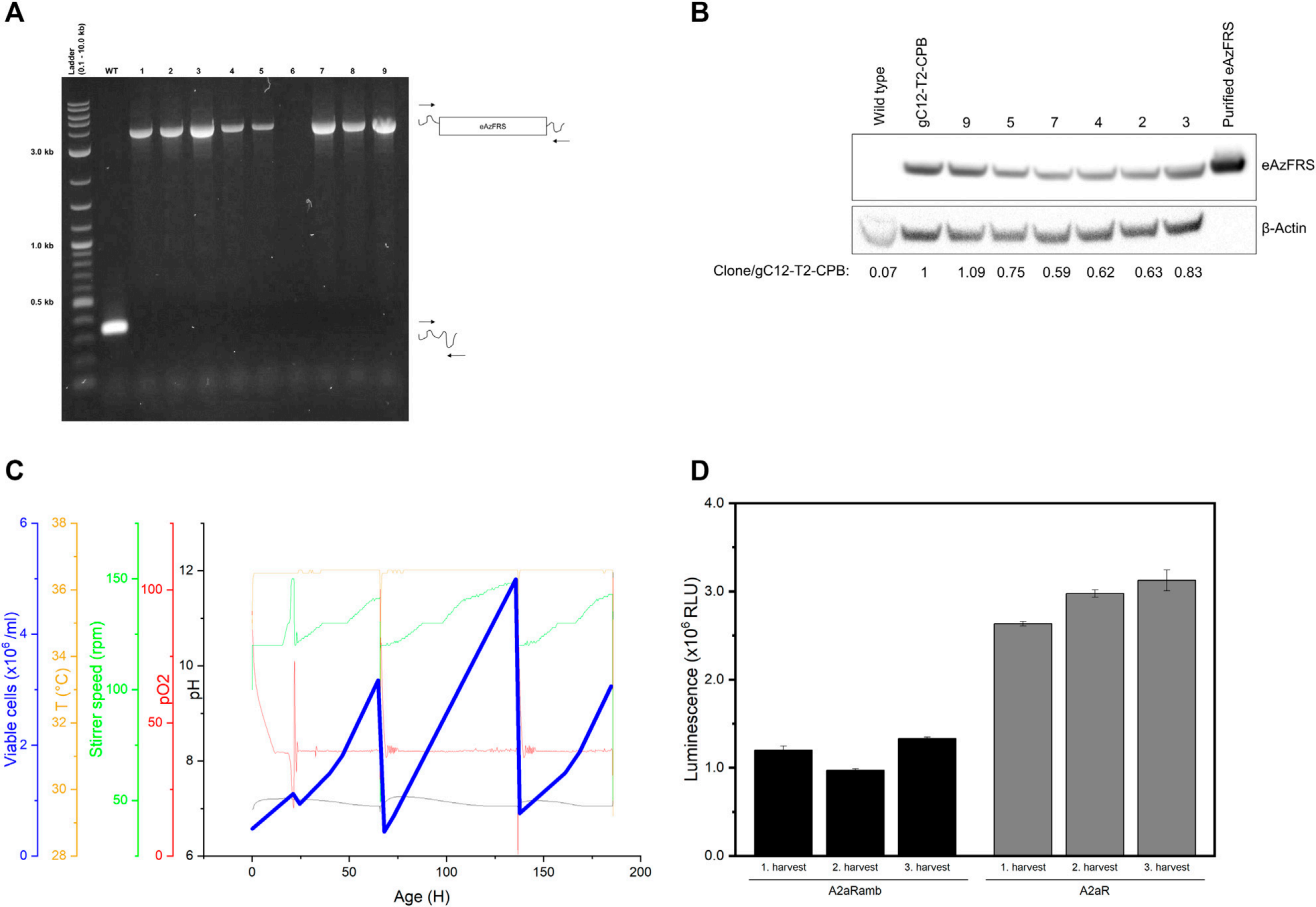
Evaluation of clonal cell lines expressing the eAzFRS. **(A)** Genotyping PCR of clonal cell lines isolated from clone pool gC12-T2-CPB. The expected outcome is indicated by visualize primer binding (arrows) on wilde type (WT) and modified cells. A Quick-Load 2-Log DNA Ladder 0.1–10.0 kb (New England Biolabs) was used. **(B)** Semi-quantitative western blot of cell clones. eAzFRS band intensities were normalized to the internal β-Actin control. The indicated fold change is expressed as ratio of band intensity of the samples compared to clone pool gC12-T2-CPB. **(C)** Exemplary fermentation process of clone RS7. Cell harvesting was performed three times at highest peaks (blue line). **(D)** Luciferase assay of cell-free synthesized A2aR and A2aRamb constructs based on cell lysates prepared from three harvest points of a cultivation process using CHO clone RS7. Experiments were performed in technical triplicates and bars represent the standard deviation.

On the one hand, working with a heterogeneous pool of edited cells provides a time saving method to produce cell lysates containing an endogenous orthogonal aaRS. On the other hand, reproducibility is a main issue. As a consequence, our objective was to create a stable cell line to overcome clonal variability of edited cell pools. Nine single clones were isolated from clone pool gC12-T2-CP-B. Genotyping PCR with primers flanking the site of integration revealed only a short PCR product for WT cells, as expected (Figure 5A). Upon successful integration of the eAzFRS expression cassette an increase of PCR product size was anticipated. The clonality of eight produced cell lines could be demonstrated due to the presence of a PCR-product at the expected size, while clone 6 showed no PCR product. Mono-allelic knock-in of the eAzFRS expression cassette can be excluded, since only one defined PCR product correlating to the exact size of the expression cassette was observed. The eAzFRS CHO clones (RS) 2–5, 7 and 9 were subjected to semi-quantitative western blot analysis, since increased outgrowth was observed compared to RS1 and RS8. A 1.09-fold increase in band intensity was observed for clone RS9 compared to the clone pool gC12-T2-CPB, while clone RS7 showed the lowest band intensity (Figure 5B).

Recombinant protein expression is often associated with a metabolic burden on the cells, leading us to compare unmodified CHO cells (wild type) with CHO clones with the highest (RS9) and lowest (RS7) eAzFRS expression by evaluating their maximum growth rates. Therefore, growth curves were plotted (Supplementary Figure S5) and resulting growth rates were compared by a Welch’s *t*-test. The maximum growth rates of WT (0.013 h^−1^ ± 0.00080), RS7 (0.014 h^−1^ ± 0.00076) and RS9 (0.015 h^−1^ ± 0.0047) were similar and there was no significant difference determined. However, cell-free reactions based on cell lysate from clone RS9 led to a lower suppression efficiency (13%) than previously observed with the cell lysate prepared from the parental pool (Supplementary Figure S6). In contrast, higher amber suppression could be observed by using cell lysate based on clone RS7 (Supplementary Figure S7). Further examination of RS7 cells by producing cell lysates highlighted their performance during the fermentation process in a 1 L bioreactor and in orthogonal cell-free reactions. In particular, it was analyzed if the absence of puromycin during the fermentation process affects the stability of eAzFRS gene expression. Consequently, we set out to cultivate clone RS7 under controlled conditions for nine days and CHO cells were harvested after three, six and nine days of cultivation as depicted in Figure 5C. Therefore, a so-called repeated batch mode was applied, leaving CHO cells in the bioreactor after each harvest point. Cell lysates prepared at three harvest points were analyzed by cell-free synthesis of A2aRamb and A2aR to assess the functionality of the novel orthogonal system over the period of time. Using the A2aRamb construct, it was shown that no reduced enzyme activity was observed after three harvesting times (Figure 5D). General protein synthesis was also not negatively affected over the cultivation period, as indicated by using A2aR construct.

Moreover, it was shown that addition of increasing concentrations of purified eAzFRS did not elevate luminescent signals in cell-free reactions with A2aRamb using the RS7 cell lysate (Supplementary Figure S7). On the contrary, addition of 3 µM eAzFRS seems to have a negative impact on the reaction.

### Transferability of the Approach to Orthogonal PylRS

**FIGURE 6:**
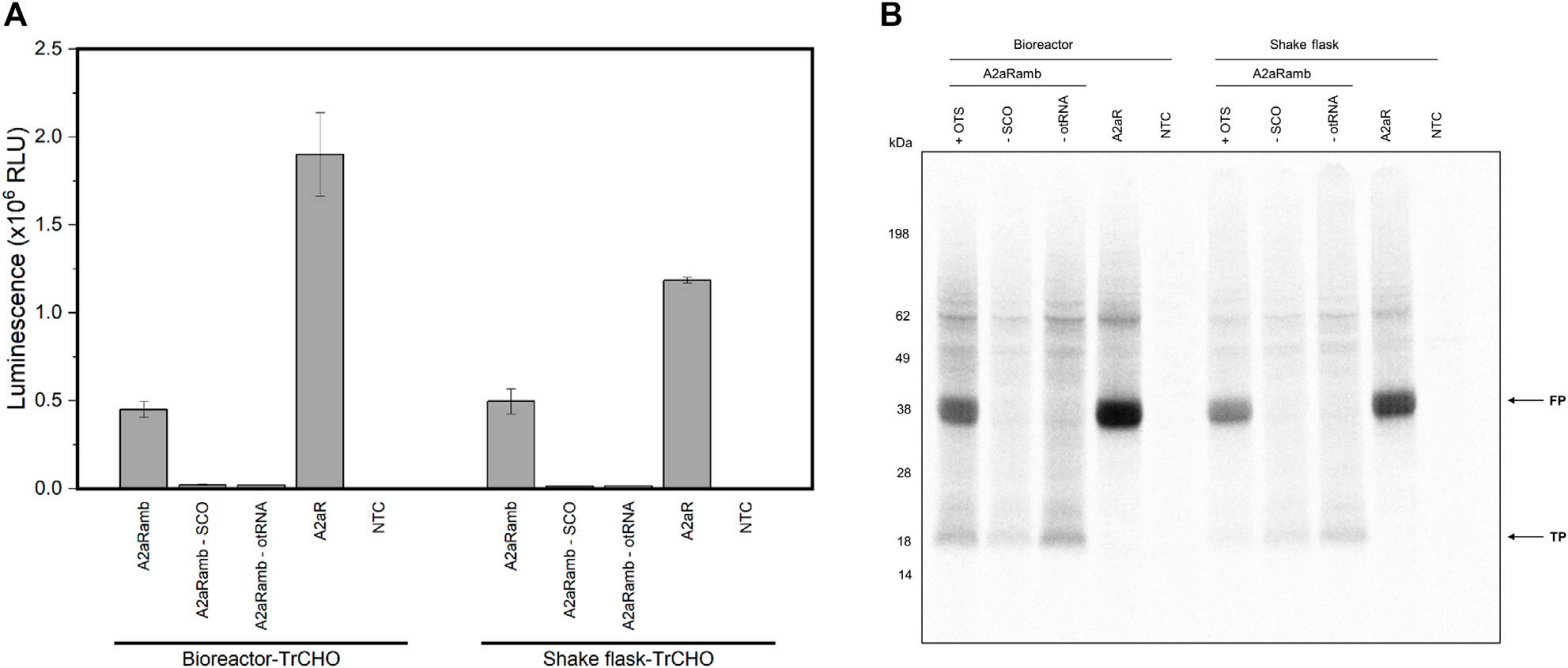
CFPS based on transiently transfected CHO cells expressing PylRS-AF. **(A)** Luciferase assay of cell-free synthesized A2aRamb and A2aR constructs based on transiently transfected CHO cells expressing the PylRS-AF. Samples with a plus indicate the addition of the particular orthogonal component, while a minus indicates the absence of the orthogonal component. Experiments were performed in technical triplicates and bars represent the standard deviation. **(B)** Autoradiography of electrophoretically separated proteins corresponding to CFPS shown in **(A)**. FP: Full-length protein, TP: Truncated protein, NTC: No-template control, SCO: Strained cyclooctyne, otRNA: Orthogonal tRNA, OTS: Orthogonal translation system, TrCHO: Transiently transfected CHO cells.

A promising orthogonal aaRS is the double mutant pyrrolysyl-tRNA synthetase (PylRS-AF), which allows large and bulky ncaa to be incorporated into proteins (Oliveira et al., 2017). A major drawback of the PylRS is the frequently observed protein instability and tendency to aggregate (Wan et al., 2014). Therefore, the intent was to check whether the PylRS can also be expressed to obtain active orthogonal aaRS in the cell lysate, thus avoiding enzyme purification and possibly accompanying enzyme inactivity. For this purpose, we transiently transfected the PylRS-AF into CHO cells and prepared cell lysate based on shake flask and bioreactor based cultivation. We could demonstrate that a suppression efficiency of 24% (bioreactor) and 42% (shake flask) was achieved by incorporating strained cyclooctyne (SCO) into the reporter construct A2aRamb (Figure 6A). These findings could be verified by autoradiography due to the presence of ^14^C leucine during the reaction (Figure 6B).

## Discussion

Chemical modification of proteins is widely used to study protein function and interactions in biological environments. Applied research can also facilitate medical diagnostics and therapy by linking drugs as well as fluorescent markers to proteins as exemplified by the production of novel antibody-drug conjugates. Suitable antibodies are often coupled to the drug *via* cysteines and lysines (Shadish and DeForest, 2020). Due to many possible coupling partners, depending on the individual amino acid composition, a mixture of conjugates results, displaying different pharmacokinetic properties (Hamblett et al., 2004; Wang et al., 2005). In this context, cell-free protein synthesis can be utilized to modify therapeutically relevant proteins by means of orthogonal systems at precisely defined positions to obtain a homogeneous mixture of antibody-drug conjugates. It has been shown that site-specifically modified Her2-binding IgG antibodies based on *E.coli* cell-free systems have been coupled to the anti-cancer agent monomethyl auristatin F, delivering the antibody-drug conjugate to the antigen (Zimmerman et al., 2014).

The orthogonal aaRS/tRNA pair is usually added to the open cell-free system. On the one hand, the simple addition of a wide variety of reactants to the translation reaction is advantageous. On the other hand, the additive can negatively affect the reaction itself, as the reaction environment is highly dependent on a defined milieu. Proteins such as aaRS are typically located in a solvent that ensures protein stability but does not correspond to the optimum of cell-free reaction conditions in terms of ion concentrations. Various studies have shown that even in the case of a minor change of ion concentrations including magnesium, potassium and chloride ions, a significant effect on protein production was observed (Brigotti et al., 2003; Spice et al., 2020; Garenne et al., 2021). In particular, a reduced amber suppression was detectable with increasing eAzFRS concentrations and thus increasing buffer concentrations. Together with the time consuming purification of aaRS, an alternative approach was achieved by Chemla et al. using cell-free systems based on *E.coli* extracts, where PylRS expression was performed prior to cell disruption (Chemla et al., 2015). Besides, commonly used *E. coli* extracts for CFPS contain the T7 RNA polymerase from T7 phage to bypass the addition of purified enzyme (Köhrer et al., 1996; Des Soye et al., 2019). In contrast to *E.coli* based systems, the present study demonstrates that orthogonal aaRS originating from *E. coli* and archaea were successfully integrated into eukaryotic cells prior to cell disruption. The resulting orthogonal translation system was used to produce the complex membrane protein A2aR in cell-free systems.

It has already been shown that orthogonal aaRS were transiently transfected to modify proteins *in vivo* (Cohen and Arbely, 2016; Meineke et al., 2020). However, it has been assumed that transfection has a negative impact on the cell lysate for CFPS as cell disruption is typically performed 2–3 days after transfection and transfection reagents can negatively affect cell viability. Contrary to expectations, CFPS based on lysates of CHO cells treated with the commonly used transfection reagent PEI were shown to have similar reporter gene activity as non-treated CHO cells.

Another aspect that was examined in the present study is the cultivation format after transient transfection prior to cell disruption. Higher reporter gene activities based on A2aR were detected with cell lysates based on cultivation using bioreactors. This could be related to the controlled cultivation conditions and thus reduced cell stress in bioreactor based cultivations. Although shake flasks are easy to use, lack of oxygen control and pH control can be challenging throughout the cultivation process (Link and Weuster-Botz, 2011). Nevertheless, satisfactory reporter gene activities could be achieved based on shake flask cultivation. Thus, with the presented orthogonal translation system, cells can be efficiently enabled for orthogonal aaRS production, cost-effective cultivation in shake flasks and subsequent cell lysate preparation for CFPS can be performed in a timesaving procedure. Of particular concern is the role of the novel orthogonal CFPS based on CHO lysate for investigating membrane proteins such as the examined A2aR, since efficient translocation of membrane proteins into microsomal structures can be achieved in certain eukaryotic cell-free systems (Brödel et al., 2014; Sonnabend et al., 2017; Zemella et al., 2017). As GPCRs account for 35% of approved drug targets, the analysis of GPCRs based on interaction studies with potential ligands is of highest interest (Sriram and Insel, 2018). Fluorescence-based interaction studies offer the possibility to identify potential ligands and inhibitors by incorporating ncaa at defined positions in GPCRs subsequently conjugating them with fluorescent molecules, thus eliminating the requirement for large fluorescent fusion proteins that may affect protein interactions and functions (Lee et al., 2019). In this context, we recently demonstrated that site-specifically fluorescently labeled A2aR was excited by the C-terminal localized Nluc after substrate addition and a signal change was measured based on the conformational change of the GPCR after addition of ligand (Zemella et al., 2019). In the present study we demonstrate that transient transfection of PylRS-AF also leads to an intact orthogonal cell-free translational system for the production of site-specifically modified A2aR. Accordingly, purification of the unstable PylRS can be bypassed, which can influence the enzyme`s activity significantly.

Cell lysates based on transient transfection showed variable amber suppression efficiencies, which could be the result of the different transfection efficiencies. Alternatively, to create a cost-effective and reproducible orthogonal cell-free system for long-term use, the eAzFRS was stably transfected into the CHO cells. In particular, stable transfection by CRISPR offers the possibility to target sites in the genome that allow high expression levels and simultaneously reduce silencing (Lo et al., 2017; Li et al., 2020). While many studies have demonstrated that *HPRT1* is suitable for high levels of antibody production in CHO cells (Wang et al., 2017; Kawabe et al., 2018), our study showed that the *C12orf35* locus is beneficial for incorporating the orthogonal eAzFRS into the CHO genome and subsequently generating active cell lysates for orthogonal translation. Disruption of the *C12orf35* gene, located in the telomeric region of chromosome 8, has been reported to affect recombinant protein production in the resulting cell lines (Ritter et al., 2016a). Using small interfering RNAs, the *C12orf35* gene was silenced and monoclonal antibody production was increased, whereas silencing of the gene and subsequent isolation of clonal cell lines resulted in fast recovery rates during the selection process. Our results are consistent with those reported by Zhao et al. who were able to generate cell lines with highest stability and anti-PD1 monoclonal antibody productivity using the *C12orf35* locus in contrast to other CHO hot spots studied such as *HPRT* and *GRIK1* (Zhao et al., 2018). However, expression of eAzFRS using the *HPRT1* locus led to lower mRNA levels and minor eAzFRS activity in cell lysates. On the one hand reduced eAzFRS transcription and production could be a result of increased epigenetic gene silencing. On the other hand *HPRT1* is a widely used target site for protein production (Wang et al., 2017; Kawabe et al., 2018). Nevertheless, the eAzFRS coding sequence is originated from *E.coli* and might have an impact on the *HPRT1* locus in CHO cells.

Addressing transcriptionally active gene loci by CRISPR ensures controlled overexpression of proteins and overcomes the unpredictable insertion of transgenes into random genomic positions. The on-target efficiency and off-target effects depend mainly on the gRNA sequence used (Wilson et al., 2018). Therefore, we designed a gRNA sequence that addresses the *HPRT1* and *C12orf35* loci and postulated that integration of eAzFRS is improved. Indeed, the novel gRNAs were shown to result in a higher eAzFRS mRNA level in comparison to the recently reported gRNA sequences (Zhao et al., 2018). This may be due to the fact that the sequence context flanking the target site and resulting gene positioning effects are of particular importance. Indeed, it has been shown that deletions in the telomeric region around *C12orf35* resulted in CHO cell lines with increased stability and high protein production rates (Ritter et al., 2016b). Epigenetic regulatory mechanisms such as DNA methylation and histone modification can strongly influence the expression level as well as gene silencing (Gibney and Nolan, 2010; Keller et al., 2019). However, it must be emphasized that off-target effects of the utilized gRNAs have not been investigated and integration of eAzFRS in other transcriptionally active gene loci cannot be excluded. In addition, mRNA expression strongly depends on the inserted gene sequence in the genomic context.

Various orthogonal aaRS would be of interest to incorporate them into CHO cells to expand the repertoire of diverse ncaa for incorporation into proteins in CFPS. In contrast, including orthogonal tRNA in the cell lysate could be challenging, since it was reported that the ratio of expression cassettes of both orthogonal tRNA and corresponding aaRS needs to be adjusted for cell-based production to achieve high suppression efficiency (Ryu and Schultz, 2006; Schmied et al., 2014). This highlights the strength of CFPS, as the tRNA can be transcribed *in vitro* followed by a titration to the cell-free system in optimal ratios.

In summary, the developed system is suitable for the incorporation of diverse orthogonal aaRS as well as other proteins of interest into the cell lysate, thus eliminating the need for the supplementation of enzymes and co-factors. As a result, a novel orthogonal eukaryotic cell-free system speeds up the production of site-specifically modified complex proteins.

## Data Availability

The datasets presented in this study can be found in online repositories. The names of the repository/repositories and accession number(s) can be found in the article/Supplementary Material.
