# Supplementary material for: Cell Engineering and Cultivation of Chinese Hamster Ovary Cells for the Development of Orthogonal Eukaryotic Cell-free Translation Systems
Source: Front Mol Biosci. 2022 Apr 14;9:832379. doi: 10.3389/fmolb.2022.832379 (PMC9109823; doi:10.3389/fmolb.2022.832379)
Supplement: Supplementary file 1 [file DataSheet1.docx]

Supplementary Material


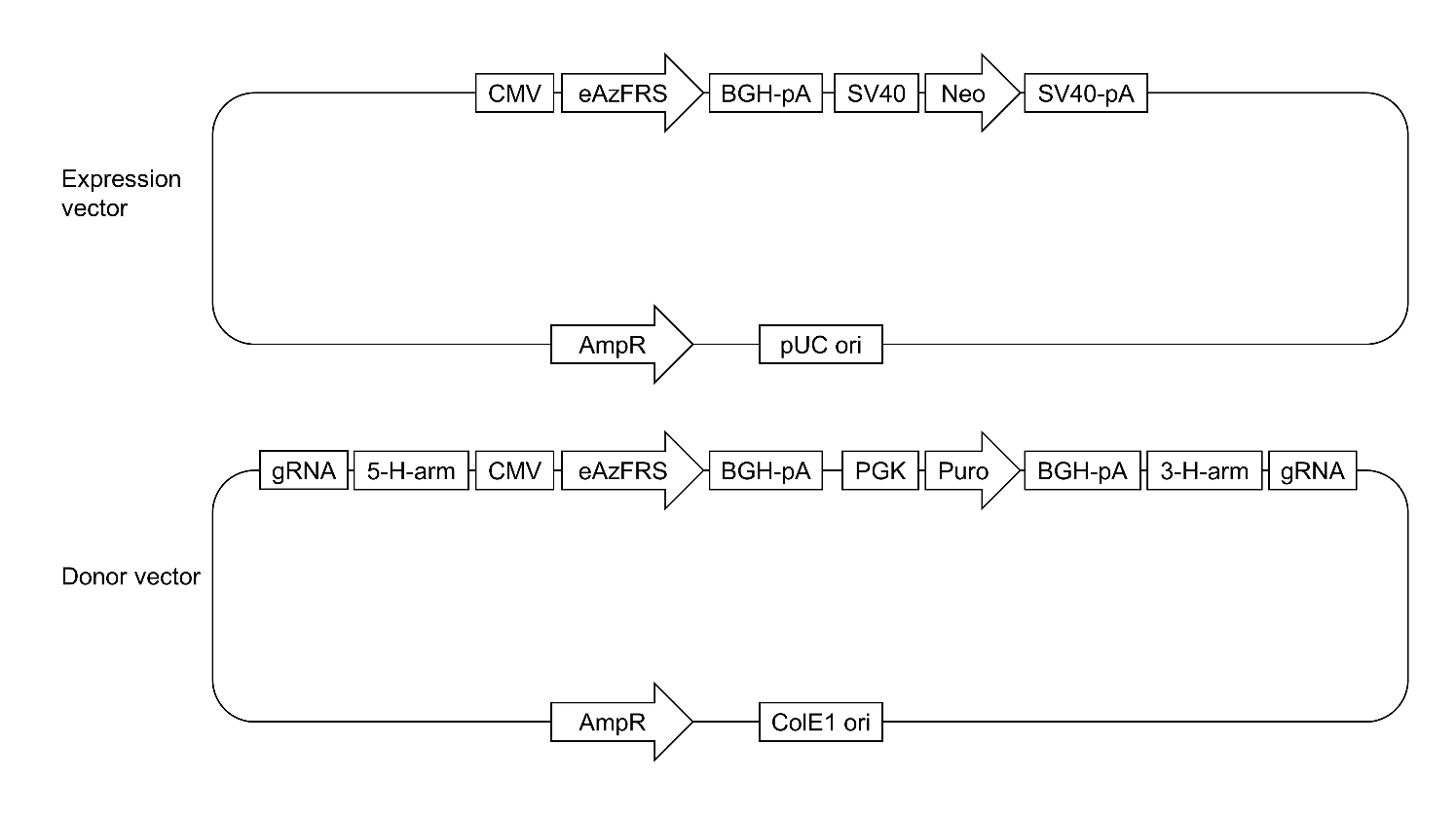


**Supplementary Figure 1: Vector illustration.** Expression vectors utilized for transient transfections are based on a CMV promoter for eAzFRS/PylRS-AF expression and a SV40 promoter for the expression of a neomycin resistance gene. Donor vectors utilized for homology directed repair based on CRISPR/Cas9 technology contain a CMV promoter for expression of eAzFRS and a PGK promoter for puromycin expression. Homology arms at the 5´-end and 3´-end are 700 bp pairs long. The donor cassette is flanked by gRNA recognition sequences to linearize the donor cassette after transfection with Cas9 and gRNA expression plasmids.





**Supplementary Figure 2: Luciferase assay of cell-free reactions based on CHO cell lysate.** Different volumes of the translation mix after a cell-free reaction with A2aR construct were analyzed by a luciferase assay to show linearity. Experiments were performed in technical triplicate and bars represent the mean ± standard deviation.

**

**

**Supplementary Figure 3: Variation of the concentration of purified eAzFRS in cell-free reactions based on CHO cell lysate.** The concentration of purified eAzFRS war varied from 0 to 5 µM to identify the optimal amount of enzyme for orthogonal translation in cell-free reactions based on CHO lysate without endogenous eAzFRS. The A2aRamb construct was utilized to analyze amber suppression using the luciferase assay. Experiments were performed in technical triplicate and bars represent the mean ± standard deviation.


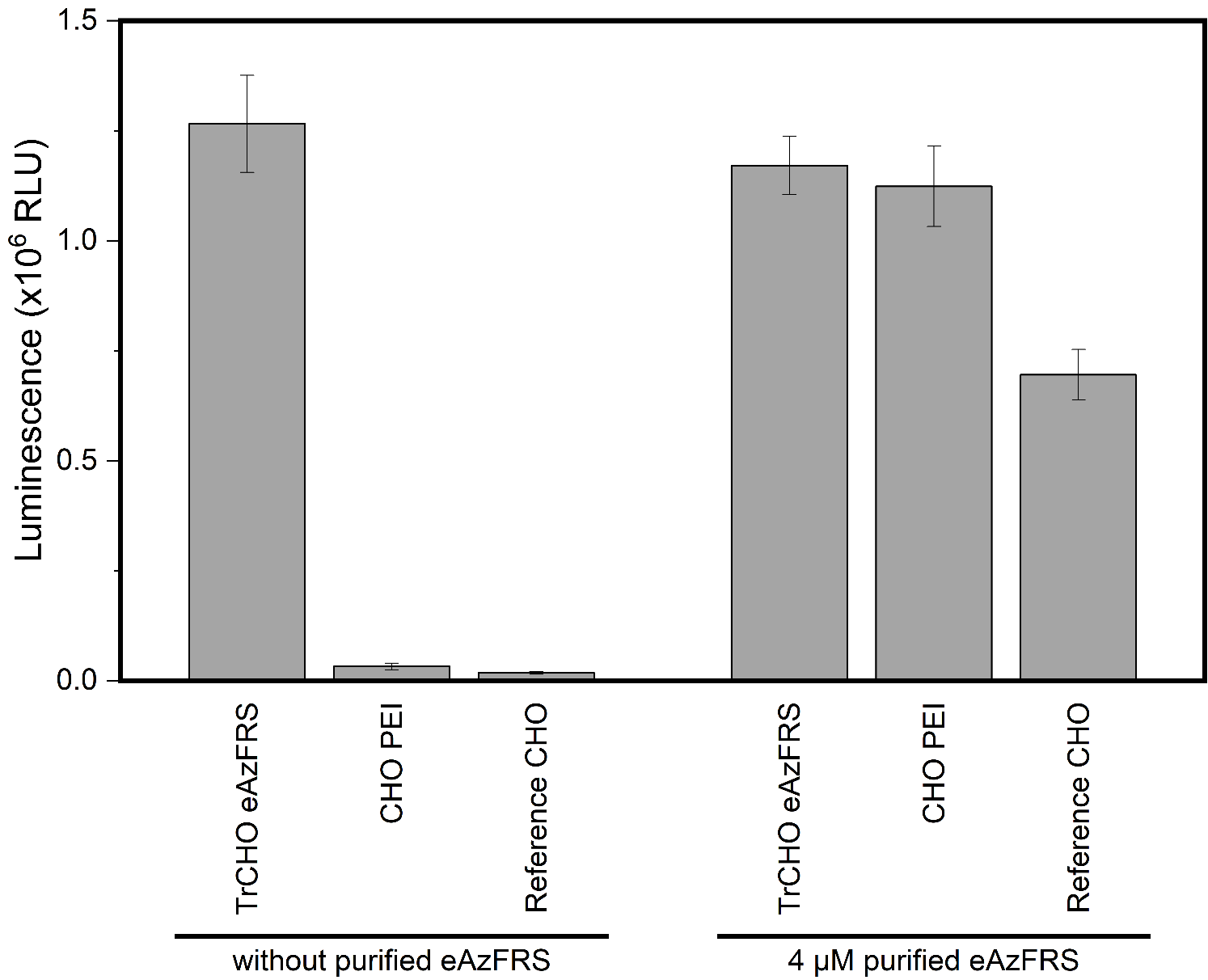


**Supplementary Figure 4: Effect of PEI on CFPS based on transiently transfected CHO cells expressing eAzFRS.** Luciferase assay of cell-free synthesized A2aRamb based on non-treated CHO cells (reference CHO), PEI treated CHO cells (CHO PEI) and transfected CHO cells with eAzFRS based on PEI (TrCHO eAzFRS). Cell-free reactions were performed in the presence or absence of purified eAzFRS. Experiments were performed in technical triplicate and bars represent the mean ± standard deviation.





**Supplementary Figure 5: Growth curves of three independent cultivations with wild type CHO (A-C), clone 7 (D-F) and clone 9 cells (G-I).**

**

**

**Supplementary Figure 6: Cell-free synthesis based on RS9.** Suppression efficiency was calculated using the A2aRamb and A2aR construct. Luminescence signals of cell-free reactions without a template (NTC) were subtracted from sample signals prior to calculation of suppression efficiency. Samples with a plus or minus indicate the addition or absence of the orthogonal tRNA (otRNA). Experiments were performed in technical triplicate and bars represent the mean ± standard deviation.

**

**

**Supplementary Figure 7: Evaluation of cell-free synthesis based on RS7.** Luciferase assay of cell-free synthesized A2aRamb and A2aR constructs based on CHO clone RS7. Concentrations of purified eAzFRS were varied from 0-3 µM. Samples with a minus indicate the absence of the orthogonal tRNA (otRNA). Experiments were performed in technical triplicate and bars represent the mean ± standard deviation. NTC: No-template control.





**Supplementary Figure 8: Fermentation of CHO cells transiently transfected with eAzFRS.**

**

**

**Supplementary Figure 9: Fermentation of CHO clone pool B (gHPRT1-CPB)**





**Supplementary Figure 10: Fermentation of CHO clone pool A (gC12-T1-CPA)**

**

**

**Supplementary Figure 11: Fermentation of CHO clone pool B (gC12-T1-CPB)**

**

**

**Supplementary Figure 12: Fermentation of CHO clone pool B (gC12-T2-CPB)**





**Supplementary Figure 13: Fermentation of CHO cells transiently transfected with PylRS.**
